# Endogenous orexin and hyperacute autonomic responses after resuscitation in a preclinical model of cardiac arrest

**DOI:** 10.3389/fnins.2024.1437464

**Published:** 2024-09-13

**Authors:** Yu Guo, Payam Gharibani, Prachi Agarwal, Hiren Modi, Sung-Min Cho, Nitish V. Thakor, Romergryko G. Geocadin

**Affiliations:** ^1^Department of Biomedical Engineering, Johns Hopkins University School of Medicine, Baltimore, MD, United States; ^2^Department of Neurology, Division of Neuroimmunology, Johns Hopkins University School of Medicine, Baltimore, MD, United States; ^3^Department of Electrical and Computer Engineering, Johns Hopkins University School of Engineering, Baltimore, MD, United States; ^4^Brain Trauma Neuroprotection Branch, Center for Military Psychiatry and Neuroscience, Walter Reed Army Institute of Research, Silver Spring, MD, United States; ^5^Departments of Neurology, Anesthesiology-Critical Care Medicine and Neurosurgery, Johns Hopkins University School of Medicine, Baltimore, MD, United States

**Keywords:** autonomic response, orexin, cerebral ischemia, heart arrest, resuscitation

## Abstract

**Objectives:**

The study of autonomic responses to cardiac arrest (CA) resuscitation deserves attention due to the impact of autonomic function on survival and arousal. Orexins are known to modulate autonomic function, but the role of endogenous orexin in hyperacute recovery of autonomic function post-resuscitation is not well understood. We hypothesized that endogenous orexin facilitates hyperacute cardiovascular sympathetic activity post-resuscitation, and this response could be attenuated by suvorexant, a dual orexin receptor antagonist.

**Methods:**

A well-established 7-min asphyxial CA rat model was studied. Heart rate (HR) and blood pressure were monitored from baseline to 90-min post-resuscitation. Autonomic function was evaluated by spectral analysis of HR variability, whereby the ratio of low- and high-frequency components (LF/HF ratio) represents the balance between sympathetic/parasympathetic activities. Plasma orexin-A levels and orexin receptors immunoreactivity in the rostral ventrolateral medulla (RVLM), the key central region for regulating sympathetic output, were measured post-resuscitation. Neurological outcome was assessed via neurologic-deficit score at 4-h post-resuscitation.

**Key results:**

A significant increase in HR was found over 25–40 min post-resuscitation (*p* < 0.01 vs. baseline), which was attenuated by suvorexant significantly (*p* < 0.05). Increased HR (from 15-to 25-min post-resuscitation) was correlated with better neurological outcomes (*rs* = 0.827, *p* = 0.005). There was no evident increase in mean arterial pressure over 25–40 min post-resuscitation, while systolic pressure was reduced greatly by suvorexant (*p* < 0.05). The LF/HF ratio was higher in animals with favorable outcomes than in animals injected with suvorexant over 30–40 min post-resuscitation (*p* < 0.05). Plasma orexin-A levels elevated at 15-min and peaked at 30-min post-resuscitation (*p* < 0.01 vs. baseline). Activated orexin receptors-immunoreactive neurons were found co-stained with tyrosine hydroxylase-immunopositive cells in the RVLM at 2-h post-resuscitation.

**Conclusion:**

Together, increased HR and elevated LF/HF ratio indicative of sympathetic arousal during a critical window (25–40 min) post-resuscitation are observed in animals with favorable outcomes. The orexin system appears to facilitate this hyperacute autonomic response post-*CA.*

## 1 Introduction

Sudden cardiac arrest (CA) affects about 437,000 cases of death in the United States annually (Tsao et al., 2023). Only 9.1% of out-of-hospital CA patients survived to hospital discharge, and 7.1% left the hospital with a functional recovery (Tsao et al., 2023). Post-CA brain injury is the common cause of morbidity and mortality (Nolan et al., 2008). CA induces global cerebral ischemia, leading to severe brain injury. However, selective vulnerability among different brain regions to CA insult exists (Nolan et al., 2008). The brainstem is less vulnerable to injury from hypoxia compared to the cortex (Geocadin et al., 2019; Brisson et al., 2014). Once dysfunction occurs in the brainstem post-resuscitation, more severe damage would be expected in the brain regions with more susceptibility (Geocadin et al., 2019). The assessment of autonomic function, such as using heart rate variability (HRV), has been utilized as a measure of brainstem integrity (Thomsen et al., 2015). Clinical research revealed that low HRV during the 24-h following ICU admission was related to mortality in critically ill patients (Bodenes et al., 2022), whereas others found that HRV measures declined in CA survivors (Huikuri et al., 1992; Dougherty and Burr, 1992). Yet few studies have investigated autonomic responses to resuscitation during the hyperacute stage post-*CA.*

The orexin system contributes to regulating autonomic functions, especially cardiovascular activity (Carrive, 2013; Grimaldi et al., 2014). The modulation effect of orexins on cardiovascular function can be triggered by various stressors, such as wakefulness and CO_2_ stress (Carrive and Kuwaki, 2017). Orexin neurons innervate multiple brain regions that modulate autonomic flow to peripheral tissues, including the rostral ventrolateral medulla (RVLM) and the medullary raphe (Carrive, 2013). The orexin-induced autonomic regulation manifests mainly in supporting sympathetic tone (Grimaldi et al., 2014). When centrally injected, both orexin-A (OxA) and orexin-B (two isopeptides of endogenous orexins) led to significant increases in heart rate (HR), mean arterial pressure (MAP), and sympathetic nerve activity in rodents (Shirasaka et al., 1999). The RVLM is a key brain area responsible for the basal and reflex control of sympathetic nerve activity in relation to the regulation of cardiovascular function (Kumagai et al., 2012). There are two types of orexin receptors (OxRs), Ox1R and Ox2R, and both of them are moderately distributed in the RVLM (Trivedi et al., 1998). Ox1R has a preferential affinity for OxA, while Ox2R exhibits similar affinities for both isopeptides of orexins (Carrive, 2013). Microinjection of OxA in the RVLM induced similar tachycardic and pressor effects in anesthetized rats (Chen et al., 2000).

Ischemic brain damage results in alterations of orexins and their receptors. In an asphyxial CA (ACA) rat model, an evident decrease of OxA concentration in cerebrospinal fluid was reported at 4-h post-resuscitation, and the administration of suvorexant, a dual orexin receptor antagonist, during the first 24-h post-CA caused severe and persistent neurological deficits (Kang et al., 2017). Upregulated mRNA levels of Ox1R were found in the medulla at 4-h post-resuscitation (Modi et al., 2017). However, the role of endogenous orexin in modulating autonomic responses to resuscitation has not been studied. We hypothesized that endogenous orexin regulates hyperacute cardiovascular responses to resuscitation from CA in a manner of supporting sympathetic activity. The aims were to investigate (a) spontaneous cardiovascular responses to resuscitation during the hyperacute stage post-CA and (b) the potential role of endogenous orexin in regulating these responses post-resuscitation.

## 2 Materials and methods

### 2.1 Animals

**Figure 1:**
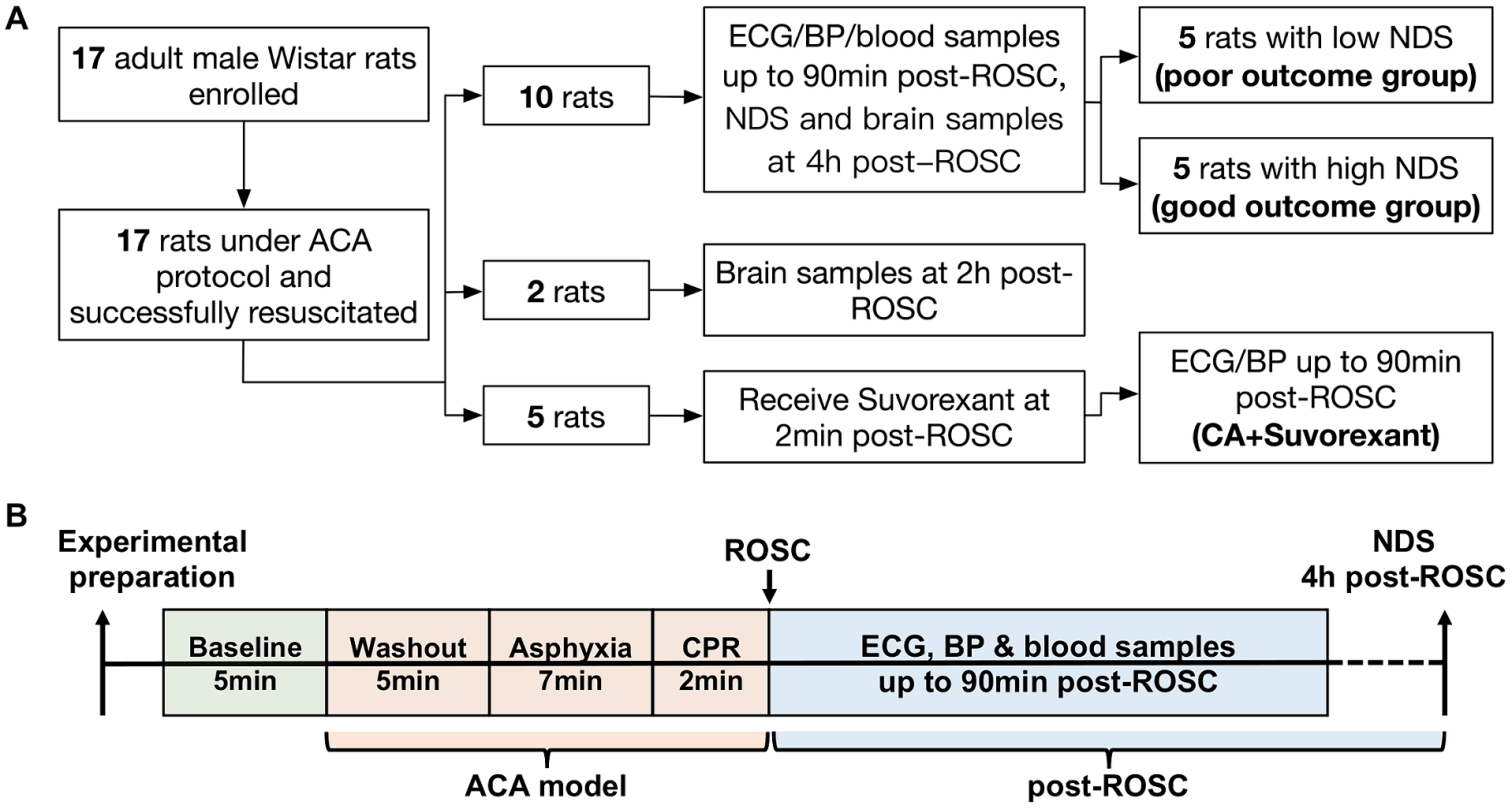
Flow diagram of the study design **(A)** and schematic diagram of the experimental protocol **(B)**.

This study was approved by the Johns Hopkins Medical Institute Animal Care and Use Committee (study number: RA19M498 Cerebral injury after cardiac arrest; approved February 28, 2020, to February 28, 2023). All procedures were conducted in accordance with the National Institutes of Health guide and reported based on the ARRIVE guidelines.^1^ A well-established ACA rat model was employed (Modi et al., 2017; Koenig et al., 2009; Wang et al., 2019; Guo et al., 2023). The sample size calculation as well as power analysis were extrapolated from our previous work. The Neurological Deficit Score (NDS) was used as the primary outcome (see Supplementary Table S1). Assuming *α* = 0.05 with a power of 0.80 and a difference of mean NDS between the two groups was 15, the minimum sample size per group was 5 subjects (Guo et al., 2023). A minimum of 5 rats (male only) per group was adequate to detect the difference in ACA-induced brain injury (Guo et al., 2023; Geocadin et al., 2000a; Wei et al., 2020; Guo et al., 2022). Here, ten rats were included to study autonomic responses to resuscitation. Two additional animals were enrolled as controls for immunohistochemistry and sacrificed at 2-h post-resuscitation (Otake et al., 2002). Another five animals were used to address the question of whether the autonomic responses could be abolished by suvorexant. The study design is illustrated in Figure 1A. All animals were Wistar male rats (~400–450 g; 11–12 weeks old; Charles River, Wilmington, MA) and subjected to 7-min ACA. In this study, the primary inclusion criteria used for ACA animals were sex (male) and age (11–12 weeks). Animals were pair-housed in a quiet environment with 12-h day/night cycles and free access to food and water.

### 2.2 Asphyxial cardiac arrest animal model

Animals were anesthetized with isoflurane (3–5% isoflurane for induction and 1.6–1.8% for maintenance delivered by 1:1 nitrogen and oxygen mixed gas) and intubated with a 16G catheter. Following intubation, the animals were placed on a stereotactic frame (Kopf Instruments, Tujunga, CA) under 1.8% isoflurane delivered via a nose cone, and three epidural electroencephalography (EEG) electrodes (Plastics One Inc., Roanoke, VA) were implanted for monitoring cortical neuronal activity (two frontal electrodes: 2 mm anterior and 2 mm lateral to bregma; one ground electrode: 2 mm posterior to lambda). Cannulation of the femoral artery and vein was then performed while the animals were ventilated via a mechanical ventilator (Kent Scientific, Torrington, CT). The ventilator was set at a tidal volume of 10 mL/kg and a positive expiratory end pressure of 3 cm H_2_O with a respiration rate of ~50 breaths per minute. Two subdermal electrodes were placed on the animals’ chest for electrocardiography (ECG) recording. HR, MAP (including systolic blood pressure, SBP and diastolic blood pressure, DBP), and EEG were monitored continuously through an RX5 TDT device (Tucker-Davis Technologies, Alachua, FL). Arterial blood gas was measured by using an i-STAT handheld analyzer (Abbott Point of Care Inc., Princeton, NJ).

After a 5-min baseline recording, ACA started with a 5-min washout period (Modi et al., 2017; Koenig et al., 2009; Wang et al., 2019; Guo et al., 2023): 2-min of 100% oxygen without isoflurane followed by rocuronium injection (2 mg/kg, I.V.; Hospira Inc., Lake Forest, IL), and then 3-min of 20% oxygen mixed with 80% nitrogen (room air). Global asphyxia was induced by stopping mechanical ventilation for a 7-min period. The achievement of CA was defined by MAP<10 mmHg and without a pulsatile-pressure wave. Cardiopulmonary resuscitation (CPR) was initiated by restarting mechanical ventilation with 100% oxygen, injecting epinephrine (7 μg/kg, I.V.; PAR Sterile Products, Rochester, MI) and NaHCO_3_ (1 mmol/kg, I.V.; Hospira Inc., Lake Forest, IL), and applying sternal chest compressions with two fingers (~200 compressions/min). Return to spontaneous circulation (ROSC) was defined as MAP>50 mmHg. After successful resuscitation, the animals were hyperventilated for about 20 min, and then the respiration rate was adjusted to maintain PaCO_2_ at 35–45 mmHg. Noted, epinephrine was only administrated once during CPR and not reinjected into the animals during post-resuscitation recording.

Post-resuscitation recording was up to 90 min. As CA-subjected animals remained comatose status (without spontaneous arousal) during this period, sedative reagents were not applied (Wei et al., 2020; Guo et al., 2022) to minimize the potential impacts of isoflurane on observations. At the end of the recording, the animals were extubated, and catheters were removed. Incisions were closed with 4–0 silk sutures. The animals were kept in a dim and quiet environment to recover.

### 2.3 Neurological outcome evaluation

The NDS has been extensively used in this ACA model to evaluate neurological outcomes (Guo et al., 2023; Geocadin et al., 2000a; Wei et al., 2020; Guo et al., 2022). The NDS scales from 0 (brain death) to 80 (healthy animal) and was assessed at 4-h post-ROSC. A score of 60 was set as the threshold for differentiating good and poor neurological outcomes (Geocadin et al., 2000b). Ten CA-subjected animals from the first cohort were separated into two groups, named the poor (NDS < 60) and good outcome group (NDS ≥ 60), for further analysis. The experimental protocol is presented in Figure 1B.

### 2.4 Enzyme-linked immunosorbent assay

Blood samples were drawn from the arterial line at baseline, 15-, 30-, 60-, and 90-min post-ROSC into tubes containing K2EDTA. The amount of blood collected each time was limited to 0.01% of the animal’s body weight. Blood samples were centrifuged at 3000 rpm for 10-min at 4°C. Plasma was then collected and stored at-80°C till further use. Plasma OxA levels were measured by ELISA kits (LifeSpan Biosciences, Seattle, WA). Absorbance was read by a microplate reader (FLUOstar Omega, BMG LABTECH Inc., Cary, NC). Samples were run in duplicate in the same assay.

### 2.5 Immunohistochemistry

We examined OxA-immunoreactivity in the RVLM and the hypothalamus, OxRs-immunoreactive (IR) neurons and tyrosine hydroxylase (TH)-immunopositive neurons in the RVLM, and Fos-immunoreactivity in the RVLM and the hypothalamus. c-Fos is a marker of tracing neuronal activity and was used here to examine the activation of OxRs-IR neurons in the RVLM at 4-h post-ROSC. c-Fos expression often peaks around 90–120 min after the activation of neurons, though the time course of c-Fos induction and decline may vary due to different inducing stimuli (Dragunow and Faull, 1989). Two more CA-subjected animals were sacrificed at 2-h post-ROSC as the control (Otake et al., 2002). Noted, we did not include a sham control (the same experimental procedure as CA-subjected animals but without induced CA) in this study because the inevitable application of anesthetics on this sham control group during the post-surgery recording period would affect the expression of these markers in the brain, making the sham condition unsuitable to compare with the post-CA condition.

The ten animals from the first cohort and the two above-mentioned animals were euthanized under deep anesthesia and perfused with a saline solution followed by 4% paraformaldehyde in phosphate buffer saline (pH = 7.3–4). The brain was extracted and post-fixed in the same fixative solution overnight at 4°C and then was kept in 10 and 30% sucrose solution consecutively at 4°C. Afterward, the brain was embedded in O.C.T. compound and sectioned serially into 40 μm thickness of coronal sections in a cryostat (Leica, Germany). For the double staining of OxA with TH or c-Fos, the slides bearing coronal brain sections were sequentially incubated with PBS containing 0.4% Triton-X and 5% normal donkey serum for 1-h at room temperature, rabbit anti-OxA (1:1,000; Millipore, Temecula, CA) with mouse anti-TH (1:200; Millipore, Temecula, CA) or mouse anti-c-Fos (1:100; Santa Cruz Biotech., Santa Cruz, CA) overnight at 4°C, and then Alexa555-conjugated donkey anti-rabbit IgG (1:1,000; Invitrogen, Waltham, MA) together with Alexa488-conjugated donkey anti-mouse IgG (1:1,000; Invitrogen, Waltham, MA) for 1-h at room temperature. Regarding the triple staining of OxRs, TH, and c-Fos, the slides were incubated for 1-h in PBS with 0.4% Triton-X and 10% normal donkey serum at room temperature, and overnight in a blocking buffer containing rabbit anti-OxRs (1:50; Bioss, Woburn, MA) and mouse anti-TH (1:200; Millipore, Temecula, CA) at 4°C, and then for 2-h in Alexa555-conjugated donkey anti-rabbit IgG (1:500; Invitrogen, Waltham, MA) and Alexa488-conjugated donkey anti-mouse IgG (1:1,000; Invitrogen, Waltham, MA) at room temperature. After 3-times PBS washes (20 min per wash), the slides were incubated in a blocking buffer with rabbit anti-c-Fos (1:2,000; Cell Signaling Technology, Danvers, MA) overnight at 4°C and then in Alexa647-conjugated donkey anti-rabbit IgG (1:1,000; BioLegend, San Diego, CA) for 1-h at room temperature. Following this step, the slides were incubated with DAPI (1,10,000) for 2 min and then mounted with an antifade mounting medium (Vector Laboratories, Burlingame, CA).

Images were acquired with a confocal laser microscope (Zeiss, Germany) and processed with Image J (National Institutes of Health, Bethesda, MD). A rat brain atlas was utilized for guidance (Paxinos and Watson, 2005). The numbers of triple-labeled neurons (stained with OxRs, TH, and c-Fos) in the RVLM (Bregman-11.80 mm to-12.80 mm) were examined in eight randomly selected sections from each of the two animals sacrificed at 2-h post-ROSC and in four randomly selected sections from each of the ten animals sacrificed at 4-h post-ROSC under 200X magnification. Positively stained neurons were counted in a blinded manner to NDS.

### 2.6 Dual orexin receptor antagonist administration

Suvorexant (Belsomra, Merck & Co., Inc., Whitehouse Station, NJ) was dissolved in a sterile saline solution at a concentration of 10 mg/mL (Fukushi et al., 2022). Five rats underwent the same 7-min ACA protocol and received intraperitoneal injection supplemented with suvorexant (30 mg/kg) within 2-min post-ROSC (Kang et al., 2017). HR and MAP (including SBP and DBP) were monitored as aforementioned. The animals were sacrificed via CO_2_ inhalation method per protocol after the recording.

### 2.7 Heart rate variability analysis

HRV analysis was conducted to quantify the autonomic function (Shaffer and Ginsberg, 2017). As described in our prior work (Guo et al., 2023), raw ECG was filtered with a second-order Butterworth notch filter to eliminate 59–61 Hz frequency and fit the target sample rate of 939 Hz. R peaks were detected by an adaptive threshold automatically. RR intervals were defined as the time intervals between two consecutive R peaks. Power spectral density was used for HRV analysis (Akselrod et al., 1981) and obtained by the Fast Fourier Transform-based Welch’s periodogram method. To calculate Welch’s periodogram, a Hann window with a 50% overlap was applied to the RR interpolated at 10 Hz. Frequency-domain measurements extracted from the power spectral density estimated for each frequency, including the relative power of very low frequency (VLF, 0–0.04 Hz), low frequency (LF, 0.04–0.15 Hz), and high frequency (HF, 0.15–0.4 Hz). LF and HF components were converted to normalized units by dividing their respective magnitudes by total power, leaving out the VLF power. LF and HF components are considered the measures of sympathetic and parasympathetic activity, respectively (Eckberg, 1997).

### 2.8 Statistical analysis

Data were analyzed by Graph Pad Prism 9.2 version (Graph Pad, San Diego, CA). Shapiro–Wilk test was employed for the normality test. Unpaired t-tests were chosen for comparisons between two groups. Repeated measures of 1−/2-way ANOVA with Bonferroni’s correction were performed for comparisons among temporal changes and multiple groups, whereby the multiplicity adjusted *p* values were reported accordingly. Spearman’s rank correlation was applied to compute correlations. The data were presented as mean ± standard deviation (SD). The statistical significance was set as *p* < 0.05.

## 3 Results

### 3.1 Neurological deficits after cardiac arrest

In the first cohort, the ten animals manifested certain degrees of neurological deficits at 4-h post-ROSC. NDS ranged 52–73 (62.70 ± 7.96; Supplementary Table S2). Using NDS = 60 as the threshold (Geocadin et al., 2002), the ten animals were divided into the poor (NDS < 60, *n* = 5) and good outcome group (NDS ≥ 60, *n* = 5). There was no significant difference between the two groups in body weight (poor vs. good: 416.2 ± 24.2 g vs. 421.6 ± 20.0 g, *p* > 0.05), preparation time (172.8 ± 14.7 min vs. 161.4 ± 7.8 min, *p* > 0.05), time to CA (241.8 ± 24.1 s vs. 260.4 ± 22.0 s, *p* > 0.05), and time to ROSC (68.2 ± 20.9 s vs. 57.2 ± 13.5 s, *p* > 0.05).

### 3.2 Acute cardiovascular responses to cardiac arrest resuscitation

**Figure 2:**
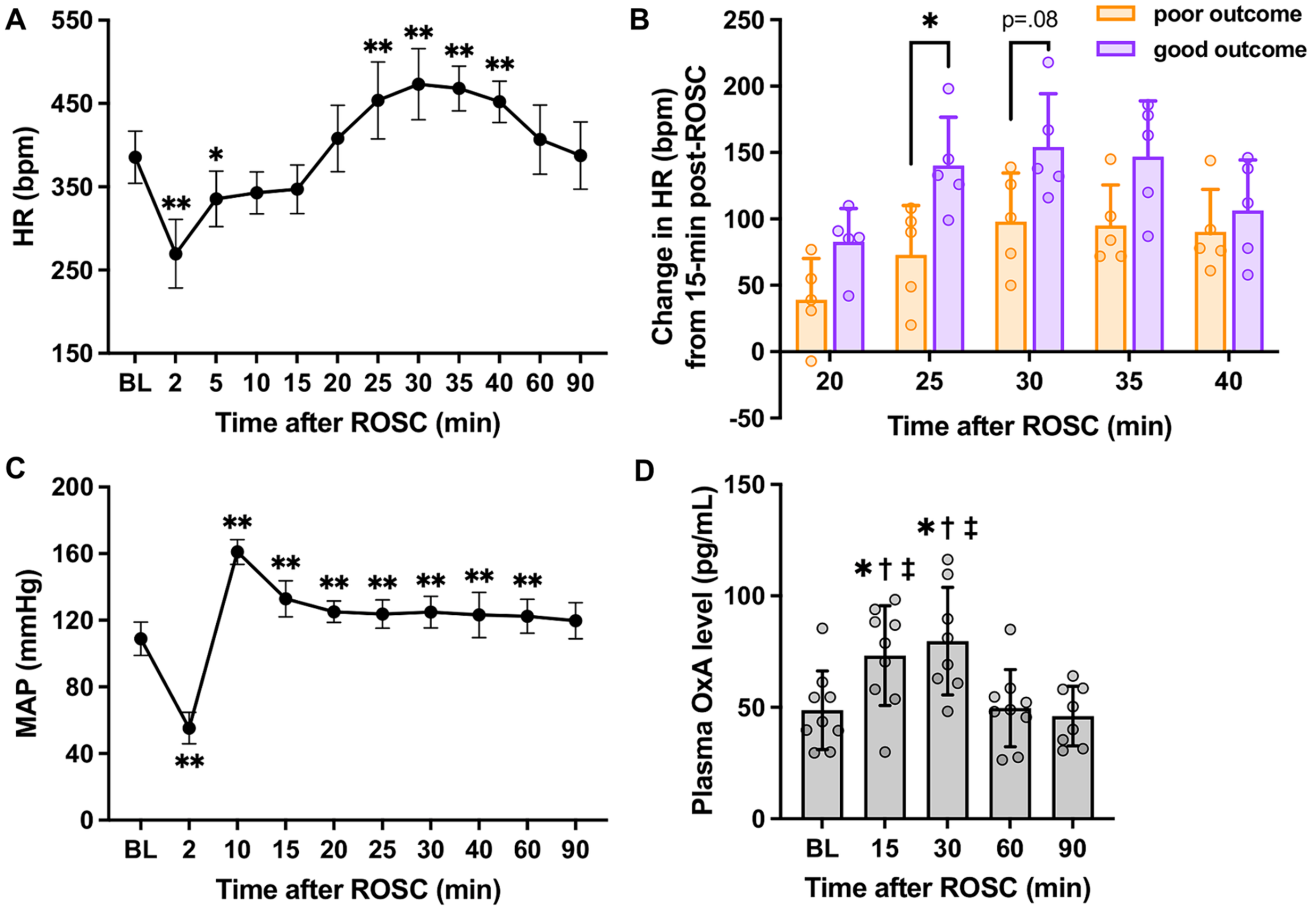
HR, MAP, and plasma OxA changes following cardiac arrest resuscitation. **(A)** HR dynamics after resuscitation (*n* = 10; RM 1-way ANOVA with Bonferroni test). * vs. baseline, *p* < 0.05; ** vs. baseline, *p* < 0.01. **(B)** The relative increase in HR in two outcome groups (*n* = 5 per group; RM 2-way ANOVA with Bonferroni test). * vs. poor outcome, *p* < 0.05. **(C)** MAP changes post-resuscitation (*n* = 10; RM 1-way ANOVA with Bonferroni test). * vs. baseline, *p* < 0.05; ** vs. baseline, *p* < 0.01. **(D)** Changes in plasma OxA levels after resuscitation from cardiac arrest. (*n* = 9; RM 1-way ANOVA with Bonferroni test). * vs. baseline, *p* < 0.05; ^†^ vs. 60-min post-ROSC, *p* < 0.05; ^‡^ vs. 90-min post-ROSC, *p* < 0.05. Data present as mean ± SD.

As shown in Figure 2A, there was a significant increase in HR over 25–40 min post-ROSC (*p* < 0.01 vs. baseline, respectively; *p* < 0.01 vs. 15-min, respectively; *n* = 10) and reached the peak at 30-min post-ROSC (473.20 ± 42.78 beats/min at 30-min vs. 385.50 ± 31.43 beats/min at baseline, *p* < 0.01). The HR at 15-min post-ROSC appeared to be the starting point of a series of significant HR changes during the first hour following resuscitation. Therefore, we chose to use the HR at this time point to further investigate the relative change in HR over the hyperacute stage post-ROSC. The relative increase in HR (compared to 15-min post-ROSC) was significantly higher in the good outcome group than in the poor outcome group at 25-min post-ROSC (140.20 ± 36.45 beats/min vs. 73.00 ± 37.16 beats/min, *p* = 0.02; Figure 2B), while an overall significant difference in this relative increased HR was also found between the two outcome groups over 20-to 40-min post-ROSC (*p* = 0.04, good vs. poor outcome group). The increased HR (from 15-to 25-min post-resuscitation) showed a strong correlation with higher NDS (*rs* = 0.827, *p* = 0.005). The increased HR from 15-to 30-min post-ROSC was also positively correlated to higher NDS, confirming good neurological outcome (*rs* = 0.663, *p* = 0.042). Besides, the relative changes in HR when compared to 10-min post-ROSC were also investigated. As shown in Supplementary Figure S1, the relative change in HR (compared to 10-min post-ROSC) in the good outcome group was evidently increased compared with the poor outcome group over 20-to 40-min post-ROSC, though a statistically significant difference between the two groups was not revealed (*p* > 0.05).

However, a similar pattern was not identified in MAP (vs. 15-min: *p* > 0.05, respectively; Figure 2C). Instead, a remarkable elevation of MAP was noted at 10-min post-ROSC. No significant differences in MAP between the two outcome groups were found over the first 90-min post-ROSC (*p* > 0.05, respectively).

### 3.3 Increased plasma orexin-A levels following resuscitation

Figure 2D illustrates that plasma OxA levels at 15-min (73.18 ± 22.33 pg./mL) and 30-min (79.73 ± 24.12 pg./mL) post-ROSC were elevated significantly compared to baseline (48.71 ± 17.62 pg./mL), 60-min (49.66 ± 17.32 pg./mL), and 90-min (46.08 ± 13.35 pg./mL) post-ROSC (*p* < 0.02, respectively), suggesting there was a surge of OxA secretion from central nervous system to the peripheral circulation during the hyperacute stage post-resuscitation.

### 3.4 Orexin receptors expressed in tyrosine hydroxylase-positive neurons in the RVLM after resuscitation

**Figure 3:**
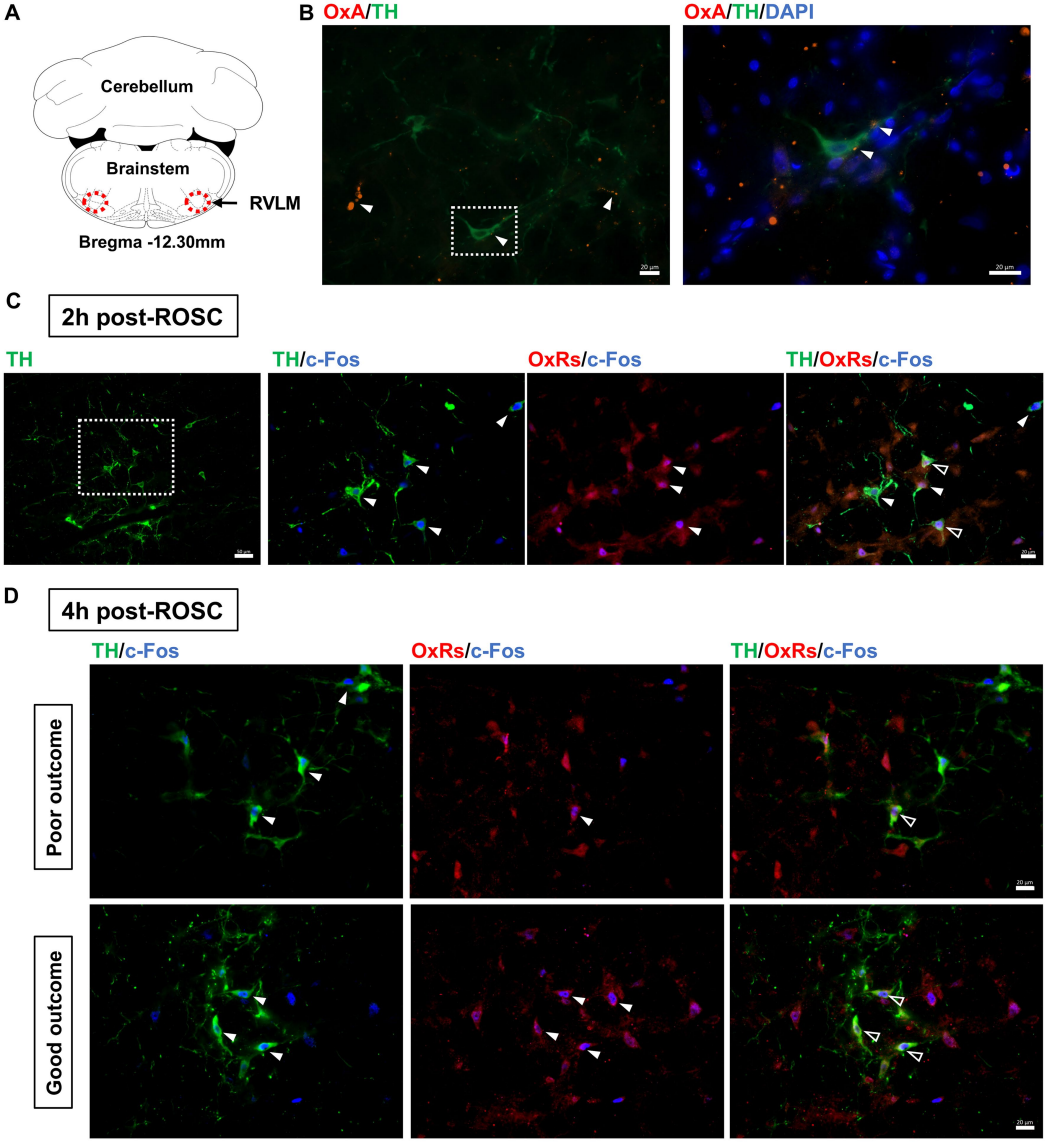
Expression of OxA and OxRs in the RVLM following resuscitation. **(A)** Schematic diagram of the anatomical position of images. Adapted from the rat brain atlas (Paxinos and Watson, 2005). **(B)** OxA-IR together with TH-IR in the RVLM at 2-h post-ROSC. White arrows denote OxA-IR terminals, and the white square shows the region of interest enlarged in the following image. Scale bar = 20 μm. Expression of OxRs, TH, and c-Fos in the RVLM at 2-h post-ROSC **(C)** and 4-h post-ROSC **(D)**, respectively. The white square indicates the region of interest that is enlarged in the following images. White arrows denote TH-IR or OxRs-IR neurons, while hollow arrows show co-stained OxRs-IR/TH-IR neurons. Scale bar = 50 μm (under 200X magnification) and 20 μm (under 400X magnification), respectively.

The expressions of OxA and TH, a marker for sympathoexcitatory catecholamine-containing neurons (Shahid et al., 2012), in the RVLM were examined at 2-h post-ROSC. OxA-IR fibers were found commonly throughout the RVLM, which was consistent with previous research (Peyron et al., 1998), and OxA-IR terminals were closely located to TH-IR cell bodies and dendrites (see Figures 3A,B).

**Figure 4:**
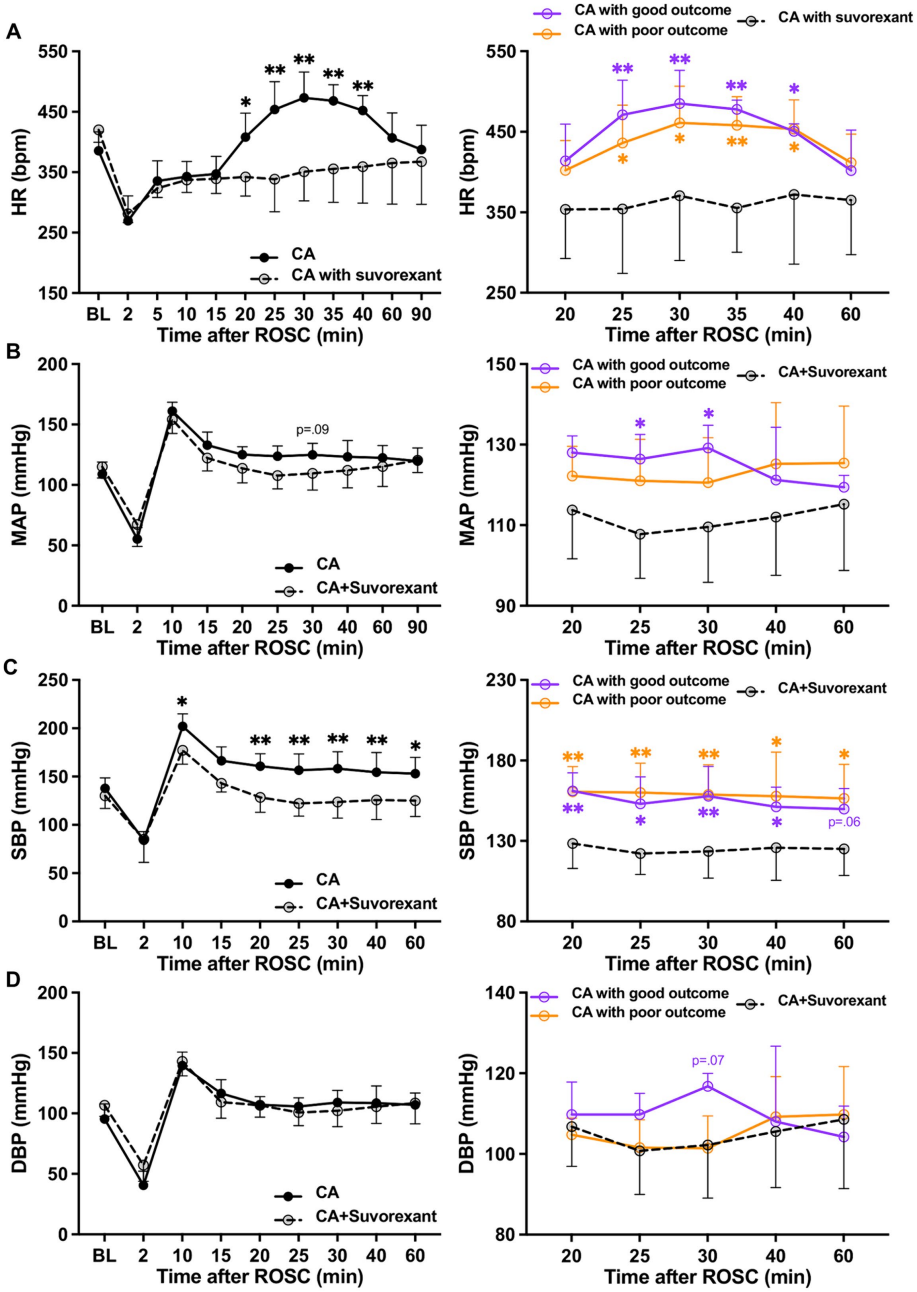
Changes in HR and blood pressure after suvorexant administration following resuscitation. **(A)** HR, **(B)** MAP, **(C)** SBP, and **(D)** DBP dynamics after suvorexant administration following cardiac arrest resuscitation (RM 2-way ANOVA with Bonferroni test). Data present as mean ± SD. * vs. CA with suvorexant, *p* < 0.05; ** vs. CA with suvorexant, *p* < 0.01.

OxRs (including Ox1R and Ox2R) were expressed in the RVLM when examined at 2-h (Figure 3C) and 4-h (Figure 3D) post-ROSC. Immunoreactivity for OxRs was observed on cell bodies and dendrites throughout the RVLM (Figures 3C,D). Immunoreactivities for OxRs and TH were found co-stained with c-Fos in the RVLM neurons post-resuscitation (Figures 3C,D). At 2-h post-ROSC, OxRs were expressed in 75 ± 13% of both TH-IR and Fos-IR neurons in the RVLM (see Table 1; raw data is available in Supplementary Table S3). The number of TH-IR neurons that co-stained with c-Fos in the RVLM was reduced from 8.19 ± 2.04 per section at 2-h post-ROSC to 5.48 ± 2.32 per section at 2-h later. The numbers of TH-IR/Fos-IR neurons and OxRs-IR/TH-IR/Fos-IR neurons in the RVLM were significantly higher in the good outcome group than in the poor outcome group (*p* < 0.05, respectively; Table 1).

**Table 1:** Numbers of OxRs-immunoreactive neurons in the RVLM after cardiac arrest resuscitation.

Group (Number of CA-subjected rats)		TH+/c-Fos + neurons (per section)	OxRs+/TH+/c-Fos + neurons (per section)	% of OxRs + in TH+/c-Fos + neurons
2-h post-ROSC (*n* = 2)		8.19 ± 2.04	6.19 ± 1.97	75 ± 13%
4-h post-ROSC	poor outcome (*n* = 5)	4.95 ± 2.35	3.45 ± 1.93	72 ± 20%
	good outcome (*n* = 5)	6.55 ± 1.93*	4.90 ± 1.65*	76 ± 17%

CA, cardiac arrest; OxRs, orexin receptors; ROSC, return to spontaneous circulation; RVLM, rostral ventrolateral medulla; TH, tyrosine hydroxylase. Data present as mean ± SD. * vs. poor outcome group, *p* < 0.05; unpaired *t*-test.

### 3.5 Effects of dual orexin receptor antagonist on cardiovascular responses to resuscitation

Suvorexant is the first dual orexin receptor antagonist approved by the Food and Drug Administration in the United States for treating insomnia (Rhyne and Anderson, 2015). We used this drug to block both Ox1R and Ox2R in CA-subjected animals mechanistically. We found the administration of suvorexant (30 mg/kg) significantly decreased HR over 20–40 min (*p* < 0.05, respectively; see Figure 4A) and SBP over 20–60 min post-ROSC (*p* < 0.05, respectively; Figure 4C) compared to the CA-subjected animals without suvorexant injection. MAP was reduced significantly by suvorexant at 25-and 30-min post-ROSC only in comparison with the good outcome group (*p* < 0.05, respectively; Figure 4B). DBP was noticeably higher in the good outcome group than that in suvorexant-treated animals at 30-min post-ROSC, though statistical significance was not identified (Figure 4D).

**Figure 5:**
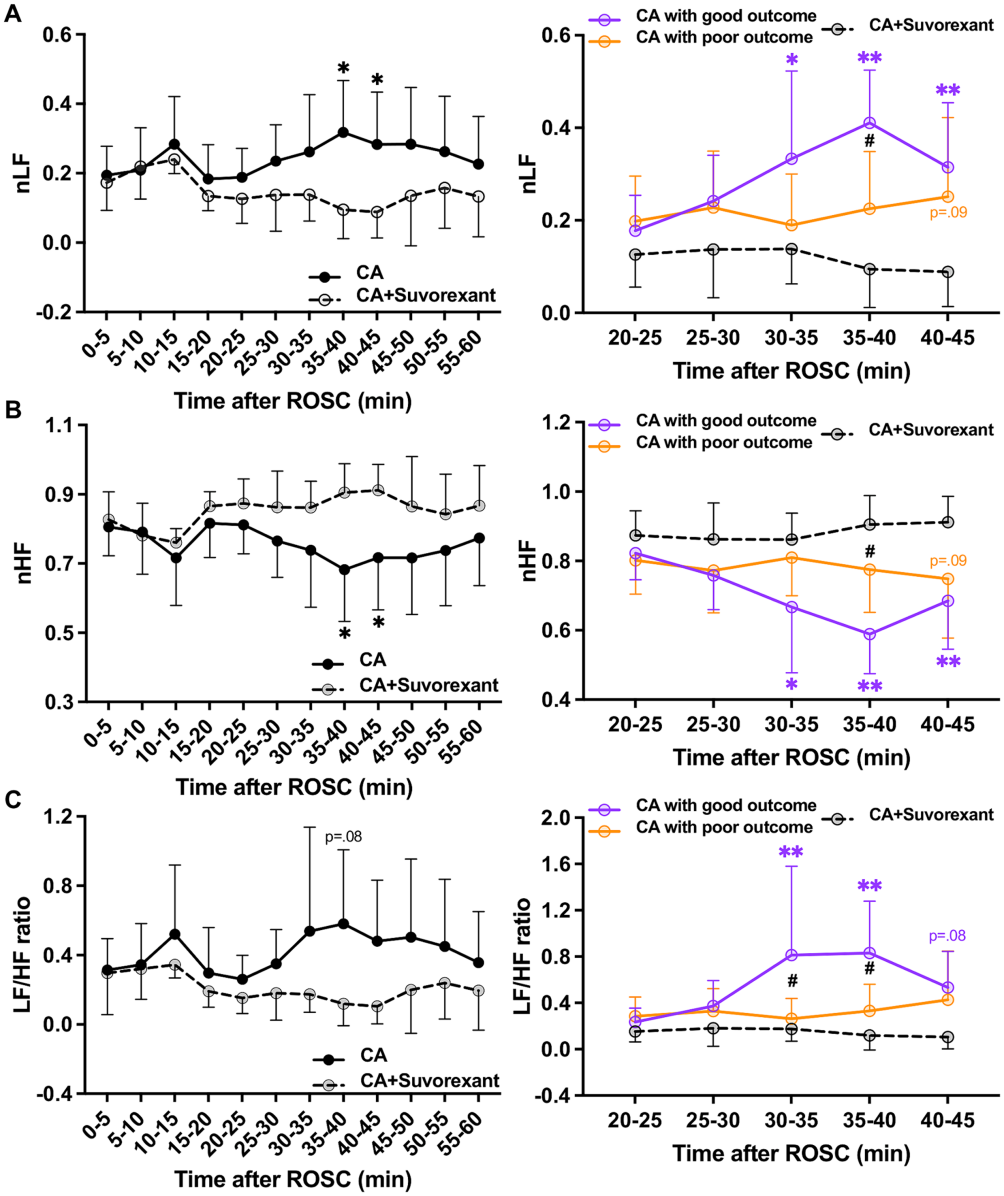
Changes in HRV parameters after suvorexant administration following resuscitation. Normalized **(A)** LF and **(B)** HF components and **(C)** LF/HF ratio after suvorexant administration following cardiac arrest resuscitation (RM 2-way ANOVA with Bonferroni test). Data present as mean ± SD. * vs. CA with suvorexant, *p* < 0.05; ** vs. CA with suvorexant, *p* < 0.01; # vs. CA with poor outcome, *p* < 0.05.

The spectral analysis of HRV was quantified at 5-min intervals during the first-hour post-ROSC (Guo et al., 2023). The LF component, indicating sympathetic tone, was significantly attenuated by suvorexant over 35–45 min post-ROSC (*p* < 0.05, respectively; Figure 5A). In comparison with the good outcome group, the LF was substantially decreased over 30–45 min post-ROSC in the suvorexant-treated group (*p* < 0.05, respectively). The HF component, reflecting parasympathetic activity, exhibited a declining trend over 20–45 min post-ROSC, which was elevated remarkably by suvorexant over 35–45 min post-ROSC (*p* < 0.05, respectively; Figure 5B). The LF/HF ratio, revealing the balance between sympathetic and parasympathetic activities, followed the LF dynamics (Figure 5C). The LF/HF ratio was significantly increased over 30–40 min post-ROSC in the good outcome group compared to the suvorexant-treated and poor outcome group, respectively (*p* < 0.05, respectively).

## 4 Discussion

In this study, we found significant increases in HR and sympathetic activity during the hyperacute stage post-resuscitation, but this reaction could be attenuated by suvorexant, especially when compared to CA-subjected animals with good outcomes. We also found that the increased HR has a strong correlation with higher NDS, while more activated catecholamine-containing neurons that expressed OxRs in the RVLM were identified in CA-subjected animals with favorable outcomes. Our results suggest endogenous orexin appears to facilitate an increase of cardiac sympathetic activity during the hyperacute stage post-ROSC, and further, this transient surge of sympathetic tone can be correlated to favorable neurological recovery after *CA.*

We studied time-dependent changes of endogenous orexins during the first hour post-resuscitation and found that plasma OxA levels dramatically increased and peaked at 30-min post-ROSC. Interestingly, HR surged remarkably post-resuscitation, with a peak around 30-min post-ROSC, which seemingly mirrors the endogenous OxA dynamics. The orexin/receptor pathway engages in the central regulation of cardiovascular function (Grimaldi et al., 2014). In this study, a strong correlation was revealed between increased HR and NDS, and more activated catecholamine-containing (TH-IR) neurons that expressed OxRs in the RVLM were found in CA-subjected animals with better NDS. Further, suvorexant significantly attenuated the increase of LF/HF ratio over 30–40 min post-ROSC compared to CA-subjected animals with favorable outcomes. These findings indicate that a transient elevated sympathetic tone, which is regulated by the orexin/receptor pathway, at the hyperacute stage post-ROSC can be linked to favorable neurological recovery.

Notably, HR and MAP did not follow the same dynamic pattern during the first hour post-resuscitation. Instead, a significant MAP elevation was found at 10-min post-ROSC and appears to be embedded in the reactive hyperperfusion phase post-CA, which usually occurs during the first 20-min post-ROSC in CA-subjected animals (Iordanova et al., 2017). This dramatic MAP increase is presumably induced by vigorous and prolonged contraction of vascular smooth muscle. It is possible that the responding receptors, such as adrenergic receptors, might have been extensively exhausted over this event, leading to different dynamics of HR and MAP. SBP was significantly decreased after blocking orexin receptors in CA-subjected rats. Basal arterial blood pressure was found to be remarkably lower in orexin knockout mice than in wild-type mice, and this difference could be canceled by α-adrenergic blockade or ganglion blockade (Kayaba et al., 2003). Our results demonstrate that the orexin system plays a critical role in regulating SBP post-*CA.* It is worth mentioning that the elevated sympathetic tone over 20–40 min post-ROSC could not be caused by the administration of epinephrine during CPR, as the elimination half-life of epinephrine is around 11 min in animals when intravenously injected (Gu et al., 1999).

Orexin-producing neurons are located predominantly in the posterior hypothalamus and extend dorsally, medially, and laterally from the fornix (Peyron et al., 1998; Harris and Aston-Jones, 2006). It was suggested that orexin neurons located in the perifornical region of the hypothalamus (PeF) may participate in regulating arousal and responses to stress (Harris and Aston-Jones, 2006). Orexin-containing neurons were found co-stained with c-Fos in the PeF at 2-h post-ROSC (Supplementary Figure S2), suggesting orexinergic neurons lactated in the PeF were activated during the hyperacute stage post-resuscitation. Orexinergic neurons can be a critical target of corticotropin-releasing factor (CRF) system in integrating stress responses (Harris and Aston-Jones, 2006; Johnson et al., 2012; Winsky-Sommerer et al., 2004). Plasma corticosterone levels were increased up to 7 days after global cerebral ischemia in rats (de la Tremblaye et al., 2014). After CA, the CRF system could be activated, stimulating the release of endogenous orexins, and further, this circuit contributes to the cardiac sympathetic activation elucidated in this study. Interestingly, we did not find significant differences in plasma OxA levels between the good and poor outcome groups over the first hour after resuscitation (see Supplementary Figure S3). The relatively small sample size included in this study may be attributed to this result. However, the plasma OxA levels appeared to peak at 15-min post-ROSC in animals with favorable neurological outcomes, which was earlier than the OxA peak found around 30-min post-ROSC in the poor outcome group. Does an earlier response to resuscitation from the orexin-producing neurons play an important role in leading to a better recovery after resuscitation? This remains to be answered in the future.

Our study has limitations. Based on our validated and approved CA animal protocol (Modi et al., 2017; Wang et al., 2019), isoflurane was employed as the anesthetic in this study. While we have instituted an anesthetic washout period in our protocol, we are not able to fully rule out some potential anesthetic effects on the autonomic responses as well as the orexin levels in the animals due to the use of isoflurane. Some considerations may potentially include lower HR, MAP, and orexin at the baseline while the animals were deeply sedated. We have to balance the anesthetic use with the humane treatment of animals, but we would like this to be considered when interpreting our results. Isoflurane could also affect c-Fos expression in the brain. It was shown that 2-h isoflurane (1.25% in oxygen) resulted in a 30% reduction of c-Fos-expressing orexinergic neurons in the PeF (Kelz et al., 2008). We examined the c-Fos expression at 2-h and 4-h post-ROSC – about 2 and 4 h after the use of isoflurane, respectively,—in different brain regions of post-CA rats. Yet, it is possible that isoflurane may have a prolonged inhibition effect on the c-Fos expression in the brain following resuscitation, which needs to be studied further.

The endogenous OxA levels were tested in plasma. Usually, neurologically related blood biomarkers are considered as a proxy of biomarkers in cerebrospinal fluid. Plasma OxA levels were reported to be in good agreement with OxA concentrations in cerebrospinal fluid (Strawn et al., 2010). Yet, it is not known how OxA levels in cerebrospinal fluid change across the first hour post-resuscitation and the relationship between the peripheral and central OxA concentrations, which needs future investigation. Another limitation was that long-term neurological and survival outcomes were not assessed in this current study. This study focused on investigating the post-ROSC change of endogenous orexin and its impacts on autonomic responses and neurological outcomes during the very early stage following resuscitation. This early period was chosen as the focus when neuroprotection is still most possible. In prior work, we showed that post-ROSC administration of OxA to CA-subjected rats significantly improved short-term neurological outcomes (at 4-h post-ROSC), and NDS was evidently higher in the OxA-treated group than the control group when measured at 72-h post-ROSC (Koenig et al., 2009). It was also reported that using suvorexant during the first 24-h post-resuscitation resulted in persistent neurological deficits up to 72-h post-ROSC in CA-subjected rats (Kang et al., 2017). It is necessary to further study if post-resuscitation OxA administration could induce prompt autonomic recovery and subsequent improvement in the long-term outcomes after CA, which may lead to meaningful modifications to the management for post-CA patients. Besides, NDS was not evaluated in CA-subjected animals treated with suvorexant. It was reported that administration of suvorexant post-resuscitation resulted in severe neurological deficits in this ACA model (Kang et al., 2017). However, suvorexant is known to have a sedative effect for 6 h in rodents (Winrow et al., 2011), which makes it difficult to assess the potential effect of suvorexant on the evaluation of short-term outcomes. Thus, 4-h NDS was not evaluated in suvorexant-treated animals in this study. Moreover, plasma levels of catecholamines were not measured in CA-subjected animals during the hyperacute stage after resuscitation. Measurement of spontaneous changes in plasma catecholamines, such as the levels of norepinephrine and epinephrine, might provide useful information in detecting the alterations of autonomic function (Goldstein and Cheshire, 2018) over this critical stage post-resuscitation. Another limitation of this study was that only OxA was measured but not both orexin isopeptides, considering that orexin-B (not OxA) may exert a cardioprotective effect in heart failure models via Ox2R (Patel Vanlata et al., 2018). Our rat model involves non-heart failure, and nonetheless, it would be interesting to further explore the endogenous levels of orexin-B following resuscitation and its therapeutic effects in a non-heart failure CA model.

This preclinical study conveys the complex interaction between the nervous and cardiovascular systems in post-CA brain injury. Following resuscitation from CA, paroxysmal sympathetic hyperactivity may occur (Tahsili-Fahadan and Geocadin, 2017). The mechanisms for sympathetic activity with tachycardia, hypertension, or fever in a post-CA state remain unclarified, and goals for interventions are undefined but largely empiric. Since this phenomenon is poorly understood, is there an HR increase or sympathetic response that could be beneficial? While an unmitigated sympathetic response could be detrimental (Meyer, 2014), is there a possible benefit in allowing a sympathetic state during the hyperacute period? The translational implication of this study is strong as we provide a better understanding of the factors that contribute to the post-CA recovery. We showed that intraventricular administration of OxA led to prompt arousal in our 7-min ACA model (Koenig et al., 2009). This study now provides some mechanistic consideration of the importance of endogenous OxA on arousal, which may be related to sympathetic activation. Our current results feature the need for monitoring autonomic responses along with orexin concentration in plasma or cerebrospinal fluid post-resuscitation, which may be helpful in post-CA patients with unresponsiveness or autonomic dysfunction.

In conclusion, our study reveals that endogenous orexin appears to modulate cardiovascular sympathetic tone during the hyperacute stage post-resuscitation, and this elevated sympathetic activity is associated with favorable neurological outcomes. Our findings highlight the possible role of acute autonomic response to resuscitation, which opens the possibility of early autonomic monitoring and consideration of the orexin system in facilitating autonomic functional recovery, leading to overall improved outcomes after *CA.*

## Data Availability

The original contributions presented in the study are included in the article/Supplementary material, further inquiries can be directed to the corresponding author.

## Footnotes

^1^ https://www.nc3rs.org.uk/arrive-guidelines
