# Supplementary material for: Endogenous orexin and hyperacute autonomic responses after resuscitation in a preclinical model of cardiac arrest
Source: Front Neurosci. 2024 Sep 13;18:1437464. doi: 10.3389/fnins.2024.1437464 (PMC11427410; doi:10.3389/fnins.2024.1437464)
Supplement: Supplementary file 1 [file Data_Sheet_1.docx]

**Supplementary Material**

**Table S1.** Neurological Deficit Score (NDS) for rats.

| **A – Arousal (0 - 19)** |  |
| --- | --- |
| Alerting | Normal (10), stuporous (5), comatose (0) |
| Eye opening | Open spontaneously (3), open to pain (1), absent (1) |
| Spontaneous respiration | Normal (6), abnormal (3), absent (0) |
| **B – Brainstem function (0 - 21)** |  |
| Olfaction | For each category: present (3), absent (0) |
| Vision |  |
| Pupillary light reflex |  |
| Corneal reflex |  |
| Startle reflex |  |
| Whisker stimulation |  |
| Swallowing |  |
| **C – Motor assessment (0 - 6)** |  |
| Strength | Normal (3), weak movement (1), no movement (0)  (each side tested and scored separately) |
| **D – Sensory assessment (0 - 6)** |  |
| Pain | Brisk withdrawal (3), weak movement (1), no movement (0)  (each side tested and scored separately) |
| **E – Motor behavior (0 - 6)** |  |
| Gait coordinate | Normal (3), abnormal (1), absent (0) |
| Balance beam walking | Normal (3), abnormal (1), absent (0) |
| **F – Behavior (0 - 12)** |  |
| Righting reflex | For each category: Normal (3), abnormal (1), absent (0) |
| Negative geotaxis |  |
| Visual placing |  |
| Turning alley |  |
| **G – Seizures (0 - 10)** |  |
| Seizures | No seizure (10), focal seizure (5), generalized seizure (0) |
| Normal condition = 80; Worst outcome = 0 |  |

**Table S2.** Prearrest baseline and arrest-resuscitation variables of CA-subjected animals.

| **Rat No.** | **Body weight (g)** | **Preparation time (min)** | **Time to ACA (sec)** | **Time to ROSC (sec)** | **NDS** |
| --- | --- | --- | --- | --- | --- |
| 1 | 398 | 168 | 230 | 62 | 56 |
| 2 | 425 | 164 | 276 | 58 | 69 |
| 3 | 397 | 182 | 283 | 87 | 54 |
| 4 | 455 | 149 | 234 | 42 | 59 |
| 5 | 422 | 170 | 260 | 80 | 65 |
| 6 | 407 | 181 | 241 | 58 | 52 |
| 7 | 424 | 184 | 221 | 92 | 57 |
| 8 | 411 | 160 | 276 | 50 | 73 |
| 9 | 398 | 164 | 223 | 52 | 71 |
| 10 | 452 | 149 | 267 | 46 | 71 |

CA, cardiac arrest; NDS, Neurological Deficit Score; ROSC, return to spontaneous circulation.

**Table S3 Datasheet of OxRs-immunoreactive neurons in the RVLM after cardiac arrest resuscitation.**

| **Rat ID** | **Section No.** | **TH+/Fos+ neurons** | **TH+/Fos+/OxRs+ neurons** |
| --- | --- | --- | --- |
| RC1 | 37-S3-1 | 11 | 11 |
|  | 37-S3-2 | 9 | 6 |
|  | 37-S4-2 | 8 | 7 |
|  | 38-S1-2 | 8 | 5 |
|  | 38-S4-1 | 13 | 9 |
|  | 38-S4-2 | 6 | 4 |
|  | 39-S2-1 | 9 | 8 |
|  | 39-S4-1 | 9 | 5 |
| RC2 | 39-S3-1 | 6 | 4 |
|  | 39-S3-2 | 6 | 4 |
|  | 39-S4-2 | 7 | 7 |
|  | 40-S1-2 | 9 | 6 |
|  | 40-S3-2 | 9 | 6 |
|  | 41-S1-2 | 9 | 7 |
|  | 41-S2-2 | 5 | 4 |
|  | 41-S3-1 | 7 | 6 |
| R30 | 68-S1-1 | 7 | 6 |
|  | 68-S1-2 | 6 | 5 |
|  | 68-S3-2 | 5 | 3 |
|  | 68-S4-2 | 6 | 6 |
| R31 | 65-S1-1 | 4 | 3 |
|  | 65-S2-1 | 4 | 3 |
|  | 65-S3-1 | 8 | 6 |
|  | 65-S4-1 | 4 | 4 |
| R32 | 64-S1-1 | 4 | 3 |
|  | 64-S1-2 | 6 | 4 |
|  | 64-S2-2 | 9 | 7 |
|  | 64-S3-2 | 6 | 3 |
| R33 | 49-S1-1 | 4 | 2 |
|  | 49-S1-2 | 3 | 3 |
|  | 49-S2-1 | 2 | 2 |
|  | 49-S3-2 | 5 | 3 |
| R34 | 47-S2-1 | 6 | 3 |
|  | 47-S3-1 | 9 | 6 |
|  | 47-S3-2 | 9 | 4 |
|  | 47-S4-2 | 8 | 5 |
| R36 | 34-S1-1 | 1 | 1 |
|  | 34-S1-2 | 7 | 4 |
|  | 34-S2-1 | 5 | 2 |
|  | 34-S3-2 | 4 | 3 |
| R38 | 38-S1-2 | 1 | 1 |
|  | 38-S2-1 | 3 | 2 |
|  | 38-S2-2 | 1 | 1 |
|  | 38-S4-2 | 3 | 1 |
| R39 | 44-S1-1 | 5 | 2 |
|  | 44-S1-2 | 6 | 5 |
|  | 44-S2-1 | 8 | 6 |
|  | 44-S2-2 | 9 | 8 |
| R40 | 43-S1-1 | 6 | 5 |
|  | 43-S2-2 | 4 | 4 |
|  | 43-S3-2 | 5 | 4 |
|  | 43-S4-1 | 6 | 5 |
| R41 | 44-S2-1 | 9 | 8 |
|  | 44-S2-2 | 9 | 6 |
|  | 44-S3-1 | 7 | 7 |
|  | 44-S3-2 | 5 | 4 |

**Figure S1**

**Figure S1** **The relative increase in HR compared to 10-min post-ROSC in two outcome groups.** Compared with the poor outcome group, the relative change in HR (compared to 10-min post-ROSC) was evidently increased in the good outcome group over 20- to 40-min post-ROSC, though this difference between the two groups was not statistically significant (*p*>0.05; n=5 per group; RM 2-way ANOVA with Bonferroni test).

**Figure S2**

**
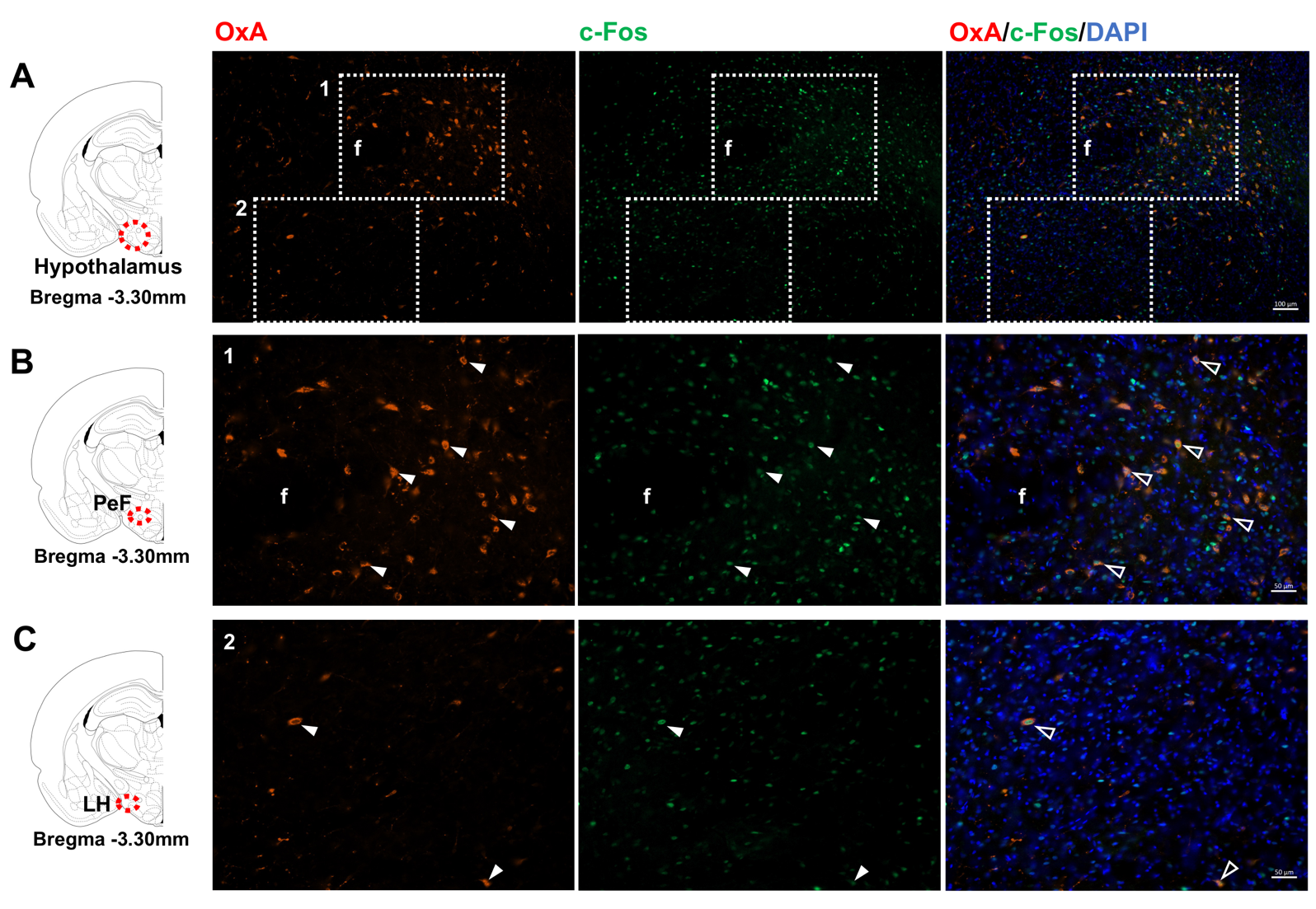
Figure S2. Expression of c-Fos in orexinergic neurons in the hypothalamus** (**A**), including the PeF (**B**) and the LH (**C**), at 2-h post-ROSC. Schematic diagrams presenting anatomical positions were adapted from the rat brain atlas (27). White squares in (**A**) indicate the regions of interest that are exhibited in (**B**) and (**C**). White arrows denote OxA-IR or Fos-IR neurons, while hollow arrows indicate co-stained OxA-IR/Fos-IR neurons. Scale bar in (**A**)=100 µm, and scale bar in (**B, C**)=50 µm. f, fornix; LH, lateral hypothalamus; PeF, perifornical region of the hypothalamus.

**Figure S3**

**Figure S3. Changes in plasma OxA levels in good and poor outcome groups after resuscitation from cardiac arrest.** There was no significant difference in plasma OxA levels between the two outcome groups over the first 90-min post-resuscitation (*p*>0.05; Mixed-effects analysis with Bonferroni test). Data present as mean ± SD. ns, not significant.
